# Characterization of In Situ Concrete of Existing RC Constructions

**DOI:** 10.3390/ma15165549

**Published:** 2022-08-12

**Authors:** Marco Vona

**Affiliations:** School of Engineering, University of Basilicata, 85100 Potenza, Italy; marco.vona@unibas.it

**Keywords:** existing RC constructions, compressive concrete strength, in situ tests, NDT–DT correlations, code provision

## Abstract

The strengths and mechanical characteristics of concrete play a key role in the safety levels for the recovery and reuse of existing RC buildings and civil engineering works. This is one of the main focuses of the current research trend. To this aim, the characteristics of concrete must be investigated: the characterization of the concrete and its in situ conditions play a key role. For these reasons, many studies on in situ and laboratory test methods and procedures have been carried out over the last two decades. In the past few years, non-destructive investigation methods have been considered reliable and used in many engineering applications, also for RC constructions. More recent codes and guidelines identify destructive test methods as a reference for practice application. However, non-destructive investigation methods can be used though exclusively in combination with destructive tests to support them. In this study, a significant database is considered to assess the reliability of the relationship between destructive and non-destructive methods for in situ concrete in existing RC constructions. The results of the analyses are used to verify the effectiveness of the methods and prediction models and suggest more effective test procedures. It can be stated that many of the existing empirical methods (based on pre-established correlations) are unable to provide a reliable evaluation of the compressive concrete strength and its variability. In practical applications, non-destructive methods often lead to unsatisfactory results for the existing reinforced concrete constructions. Finally, based on the results, some first operational indications are provided for practical investigations and future possible codes and guideline improvements.

## 1. Introduction

One of the main focuses of the current research trend is the recovery and reuse of existing RC buildings and civil engineering works. They are publicly and privately owned and are often severely degraded. Consequently, they are often underused and in many cases unused. The topic is particularly important in Europe, in which post-war reconstruction activities (after 1945) led to the construction of a very high number of RC buildings and civil engineering works. Currently, these buildings are very old and require assessment and retrofitting interventions.

**Figure 1:**
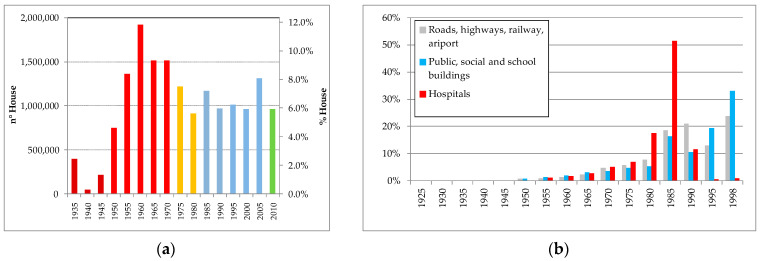
Residential buildings and main civil engineering works: (**a**) distribution according to age of the houses built; different colors refer to different Italian codes and/or new and significant seismic classification; (**b**) distribution of financial resources for engineering works that are totally or partially state-funded (source: www.istat.it (accessed on 24 January 2022)).

For example, in Italy, the RC construction heritage is among the oldest in Europe and requires substantial and extraordinary maintenance as well as functional, structural, and seismic retrofitting. The criticalities are amplified by the recent seismic classifications. As an example, in Figure 1a, a concise representation of the distribution of residential buildings and civil engineering works is reported.

However, the seismic performance assessment of existing buildings and civil engineering works is a very complex and difficult task. In recent years, the research and professional activities and the effects of earthquakes [1] have highlighted strong differences between new and existing RC structures, especially the older ones. In particular, in Italy, there is a significant number of existing RC buildings and civil engineering works. In the last twenty years, the Italian code [2] has classified almost the entire Italian territory as seismic, highlighting the need to assess the performance of existing buildings through new and specific verification methods. Moreover, in Italy, about half of the existing buildings are made of reinforced concrete. In most cases, they are designed only for gravity loads, without specific seismic guidelines [3]. Similarly, civil engineering works (infrastructures) have, in many cases, been designed without seismic and modern standards and with less effective actions than those currently provided by the codes. The existing RC buildings and civil engineering works are increasingly under assessment.

The increasing need for the use and rehabilitation of existing RC buildings and civil engineering works requires improving the assessment and investigation procedures, particularly for the execution and processing of the test results of the materials. The assessment and seismic retrofitting of the existing RC buildings has been dealt with in specific codes in many countries since the nineties [4] from which several guidelines [5], codes and recommendations [6], scientific publications [7] and consequent professional activities have derived to better explain the new trend in the codes. In Europe, the issue is considered in Eurocode 8, part 3, assessment and retrofitting of buildings [8]. The approaches of the European code [8] and those of the recent Italian code [2] are consistent with each other and were largely improved in the latest Italian code [9].

**Figure 2:**
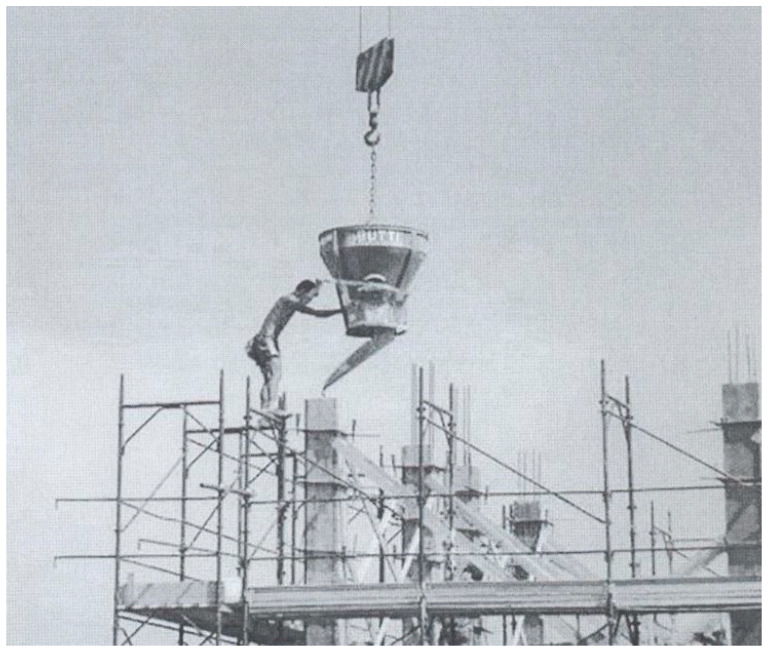
The old conventional concrete construction technology.

In the current Italian code, the part dedicated to the existing constructions is reported in chapter 8 which contains the general principles and the main issues of existing constructions; even though significant options and choices are left up to the professional’s practice. More quantitative details are reported in the guidelines [10] which address the issues that support the professionals in acquiring the quantitative and qualitative information necessary for a broader knowledge of the existing characteristics of the building. The most significant differences that the recent codes have introduced for the assessment of existing constructions are in the: (i) geometry, (ii) construction details and (iii) materials. These parameters condition the assessment step and the subsequent intervention strategy. The third element (material characterization) plays a fundamental role, especially with regard to the concrete used in reinforced concrete structures. Strong differences and variability in the mechanical characteristics of materials, in particular with regard to concrete, were verified on many existing structures [11,12,13]. The concrete is often of a lower quality than expected. Under such conditions, the most widespread testing methodologies may not be effective due to several factors. First of all, the results of the tests depend on various factors such as specific weight, water/cement ratio, aggregate/cement ratio, type of aggregate, percentage of humidity, and presence of reinforcements. These factors are well known and extensively studied (for example, [14]). Moreover, other factors that are specific to old constructions can affect the results of the tests: the age of the concrete, the original construction procedures namely pouring, compacting, curing, quality controls (Figure 2), the period of service, the effects of the degradation induced by the environment and use, the effects of damage due to earthquakes and other actions and loads.

The latest Italian code [10] has introduced major innovations to define the level of knowledge on the basis of in situ and laboratory investigations. The amount of tests to be performed is linked to the level of knowledge by means of indications that are purely indicative. Therefore, the professional has a great responsibility for the design of the surveys, the choice and application of the investigation methods, and the elaboration of the results. The Italian code identifies tests (DTs) [15] to be the most effective method to directly estimate the compressive in situ strength of concrete. Other types of non-destructive tests (NDTs) [16] could be used, considering the relationships with the results of DTs [17,18]. However, non-destructive methods can be used only and exclusively in combination with DTs. Many design codes or guidelines provide indications forseveral procedures. Nevertheless, they are often not thorough due to the complexity of the problem. A typical problem for the existing RC constructions is where the concrete may be of poor quality and the operative applications of the non-destructive methods often lead to unsatisfactory results. Consequently, the practical efforts and required resources for NDTs do not seem to be consistent with the results obtained.

The purpose of this study is to identify the critical issues in current investigation procedures and possible improvements. Similar studies have been carried out on these topics in the past. However, no alternative and corrective methods useful for the applications were clearly identified.

In particular, in this study, the operational issues of DTs and NDTs for concrete in existing RC construction, as well as the reliable interpretation of the results, are investigated.

A contribution of the study is the database considered. The database was obtained from laboratory investigations and professional practice activities. Thanks to the database, the reliability of the relationship between destructive and non-destructive methods for in situ concrete inthe existing RC constructionsis considered and assessed.

As reported in the following sections, the investigation procedures for existing RC construction based on NDTs and the commonly used NDT–DT correlations highlighted significant criticalities, particularly in their ability to investigate and estimate the characteristics of the concrete. Such methods, which are often developed in laboratories and validated for new concrete, are often not suitable for investigating the existing RC constructions. Based on the reported experimental campaigns, some possible improvements are consequently defined from the obtained results.

## 2. Methods and Materials

To define the method followed in the study and before describing the materials, it is necessary to highlight the most recent and relevant literature, although a complete review is not among the objectives of the study. For the mechanical characterization of concrete (in short, the concrete strength), the codes and guidelines consider DTs and NDTs. For existing RC construction, NDTs [2,9,10,11,19,20] must always be correlated with DTs;they cannot be used alone.As a matter of fact, NDT methods are affected by many uncertainties [21,22,23,24,25].Consequently, the relationship between DTs and NDTs is still the subject of many studies [26,27,28,29,30,31,32,33,34,35,36]. In the same way, the existing relationships between NDTs and concrete strength have been widely investigated [29,34,35,36] but should not be considered representative and simply applied for existing RC construction.They must be defined for the specific case under consideration.

In practical applications, one of the greatest difficulties is to identify the homogeneous areas of the structure in terms of concrete properties, and typically, NDT methods are used for this purpose [37]. However, each method (NDT) has variability that does not represent the mechanical characteristics of the concrete.Furthermore, the DT–NDT correlations have a high degree of uncertainty so the result is often unreliable.If this is the case, some NDT methods should also be excluded from surveys for the identification of concrete variability.Many interesting experiments were carried out [21,22,23,24,25,26,27,28,29,30,31,32,33,34,35,36,37,38] but the problem is still open, especially for the older RC structures.Uncertainties influence and can make DT–NDT correlations unusable.

The study of DT–NDT correlations (such as the SonReb method, SONic REBound) is still open and every possible contribution is still important.The SonReb method is undoubtedly the most common approach. SonReb procedures arebased on rebound number (*RN*) and ultrasonic velocity (*V*). The approachwas born in the 1970s and initially, its application was not based on direct correlation with destructive testing. In addition, with particular reference to the current application on existing structures, its use was significantly different. It was mainly dedicated to new concrete (for example, [39]). In the scientific literature, there are many studiesabout the SonReb method, with different empirical forms (i.e., based on linear, polynomial, power, exponential or logarithmic forms).

However, it must be emphasized that the most commonly used form in the last 30 years has been the exponential one where the relationship between the strength of the concrete and the NDTs is based on the form (1):

(1) $$f_{c}=a\cdot RN_{}^{b}\cdot V_{}^{c}$$

This form has been used since the 1970s but probably owes its greatest diffusion to Rilem [20]. The coefficients *a*, *b* and *c* are derived for each study with different experiments (see for example, [38]).

In this work, it was not considered necessary to report further details and indicate the various expressions and their comparisons. On the other hand, considering Italian data similar to what is contained herein, a SonReb reliability study [38] was recently carried out and an interesting review of the main forms and their comparison is reported.

More recently, some interesting applications have been made regarding a new emerging application area for artificial neural networks (ANNs) in civil engineering [40]. Some ANN-based techniques are used to study the strength of concrete by NDT [41]: these techniques are integrated with experimental results on cubic concrete specimens. The procedures have shown significant results and significant potential development by limiting negative effects of the natural dispersion of NDTs values [42].

It must be noted about the validation of the SonReb methodthat many forms were defined on laboratory-prepared samples, many even using cubic specimens [43,44,45,46,47,48,49]. In fact, muchresearch can now be considered outdated.

To this aim, the time reference could be the European code [8] and therefore carried out well before the problem of the assessment of existing RC constructions became crucial and widespread. Only more recently, some empirical expressions have been defined considering cylindrical samples extracted from existing structures (core drilling) [50]. Several alternative procedureswere proposed to obtain the relationship between in situ concrete strength and NDTs values, such as SonReb procedures, based on rebound number (RN) and ultrasonic velocity (V) for which specific coefficients are evaluated for investigated concrete [13,38,47,49].

Globally, the classic SonReb method seems inconsistent with the knowledge objectives of existing constructions. It is not consistent with the Italian code provision [10,11]. Furthermore, the overall costs associated with the investigation NDTs can still be considered high, compared with DTs for non-structural restoration elements, even more, when considered with the wide extension that is necessary for NDTs calibration, commonly considered as NDTs:DTs = 3:1.

For the above reasons, the evaluation of the mechanical characteristics of concrete (in particular, but not only, compressive strength) must be based mainly (or also exclusively) on DTs. NDTs must be considered only as a (non-essential) support for the evaluation of the homogeneity of the characteristics.Above all, the NDTs must never be used individually but only and always in conjunction with the DTs.In fact, NDTs are not satisfactory methods for the estimation of the mechanical characteristics and, in particular, the strength of concrete.These application restrictions are expressly provided for in the Italian code [10,11].

Consequently, the NDTs should not be used a priori for the rough determination of the characteristics of the concrete and neither for the a priori identification of homogeneous areas.Using them in this way could be misleading.

Nevertheless, DTs too present many uncertainties; they are linked to: (i) reliability and accuracy of the methods, (ii) characteristics and variability of the strength of the concrete in the structures, and (iii) execution of extraction tests. Conversely, some experimental programs on reinforced concrete elements showed the high within-member, within construction variability of in situ concrete strength [13,38,47,49] and the possible negative effects of core drilling on reinforced concrete columns [50], in particular for structural elements with low concrete strength. For the extracted concrete samples it must be taken into account that there are many differences between the resistance measured on the core samples and the actual insitu strength. Classically, the main considered factors [13] are the size and geometry of the cores, the coring direction, the presence of reinforcing bars or other inclusions, and the effect of drilling damage. However, age and design code of the construction, compressive stress, and management of the construction affect the results of the DTs but for these elements, there are still no reliable and univocal parameters and/or coefficients. The approach suggested in most codes based on the correlation of NDT and DT in situ results may not be reliable. Therefore, the implementation of the tests must be considered a very delicate issue.

In the practice application, design of investigation campaigns and results analyses play a key role insubsequent assessments. They are affected bythe limited number of experimental observations and thus the obtained results (in terms of concrete characteristics) could not be statistically representative ofthe entire construction or its part. Consequently, many more studies (as this study) are needed for the validation of NDTs, and any new contribution could be useful.

### Database

In the database, the samples are cylindrical samples extracted from existing RC structures by core drilling.

Moreover, some considerations regarding the usefulness of in situ NDTs are reported. The characteristics of the in situ tests are compared with the provisions of the codes in force at the time of the design and construction of the structures. It is to be noted that without this reference, the interpretation of the results of the tests would be ill-conditioned. In order to not make the study too long, further analyses of the database will be conducted in subsequent studies. As an example, the variability in the individual structures due to position, type of element, number of samples extracted, size of the structure, etc. has not been analyzed in depth. Similarly, no issues relating to the construction methods, management and degradation of the structures were developed, which also have a significant influence on the results of the tests.

The database is extremely interesting. It is derived from experimental and professional activities that were conducted or coordinated at the University of Basilicata and it considers activities carried out by other test centers and by professionals. This heterogeneous origin can be considered a valuable characteristic of the database which was created aftermany years of activities and research. The latest data were obtained in 2021. The database relates to hundreds of RC constructions (buildings and civil engineering works) located in the south-central Italian territory, over 7 regions. The RC buildings and civil engineering works were designed and used for public (infrastructures, schools, hospitals, barracks, offices, etc.) and private (mainly residential) use and were built from the 1950s. The database wascollected during several years of experimental programs and during several programs of seismic vulnerability assessments of public and private RC constructions. In these activities, the codes and guidelines for DTs and NDTs were used. Columns, walls and beams are the structural elements tested; their choice and the location of the tests were defined by the engineers, based on the construction characteristics considered.

The database contains DT and NDT results. In consideration of the main objective of this study, only the NDT cases were excluded, thus the following subsets were identified.

1. Subset DB_1: 2010 samples with compression tests ($f_{c,core}$) only (this is the main database).
2. Subset DB_2: 1039 samples with Specific Weight (SW).
3. Subset DB_3: 1175 samples with $f_{c,core}$, rebound hammer test (RN), and ultrasonic velocity test (V).
4. Subset DB_4: 202 samples with compression tests and ultrasonic velocity tests, $f_{c,core}$ and V.

**Figure 3:**
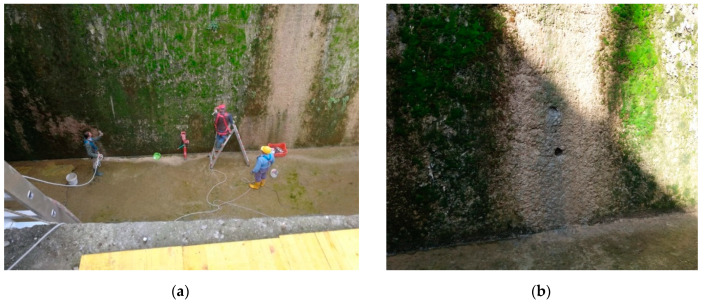
Investigation of the civil engineering works of a dam (**a**) and the current state of its concrete (**b**).

**Figure 4:**
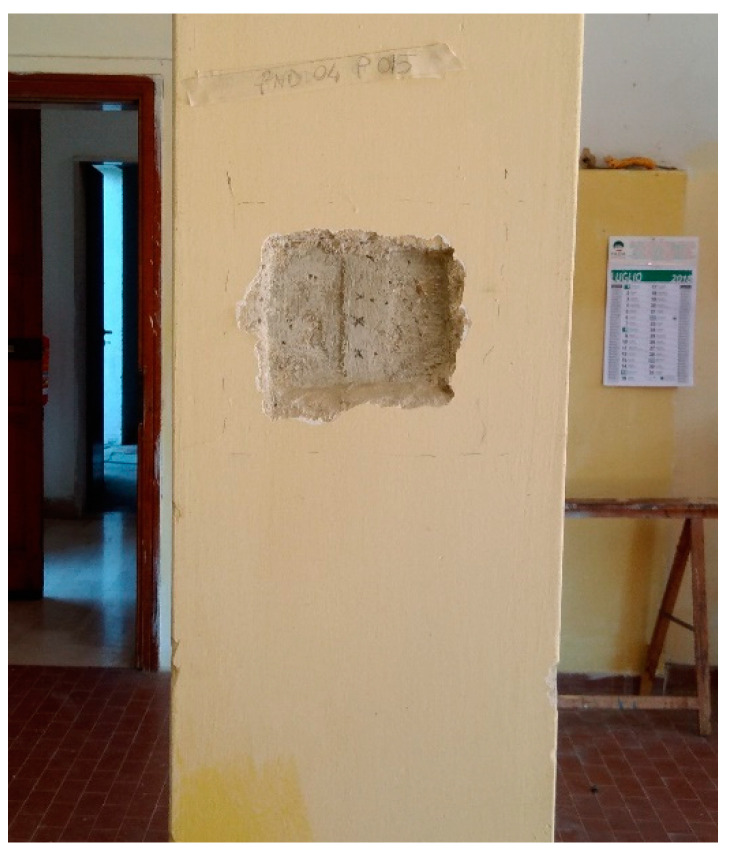
NDTs: preparation for Hammer Test and Ultrasonic velocity Test.

The difference between DB_1 and DB_3 reflects the operational difficulty of carrying out NDTs in a widespread and reliable way on existing constructions. For example, in civil engineering works the execution of reliable NDTs (in particular ultrasound) is often prevented by the construction type, shape, size, and current state of the works under investigation, for example, see the work in Figure 3 where direct velocity values are not investigable (Figure 3a) and rebound hammer test is not possible or too expensive (Figure 3b). In ordinary buildings, the execution and diffusion of NDTs areoften hindered by functional issues, for example, the presence of non-structural elements and the state of conservation and degradation of buildings. Furthermore, in these cases, the overall cost of the single NDTs (including the costs of restoring the non-structural elements, Figure 4) is comparable with DTs.

Lastly, a limited subset of samples (DB_4.1) was considered based on the results of some laboratory and in situ investigations. DB_4.1 reports the data relating to the compression tests and direct velocity values, both performed in situ (on the structural elements) and in the laboratory (on the samples, before the compression tests). The database is constantly improved and updated and possesses the characteristics shown in the following figures and tables.

The database can be considered reliable in terms of credibility and adequacy. Major errors were removed (outliers and the like). Primarily, the quality of the database can be considered consistent with the main objectives of the study. The database is based on measurements that were conducted in certified laboratories considering the same standard code.

## 3. Results

Below, each database is investigated individually. Following the main objective of the study, the main results are reported but thanks to the significant value of the database, other studies and applications will be possible in the future beyond the objectives of this work.

### 3.1. Subset DB_1: Compression Test Analyses

**Figure 5:**
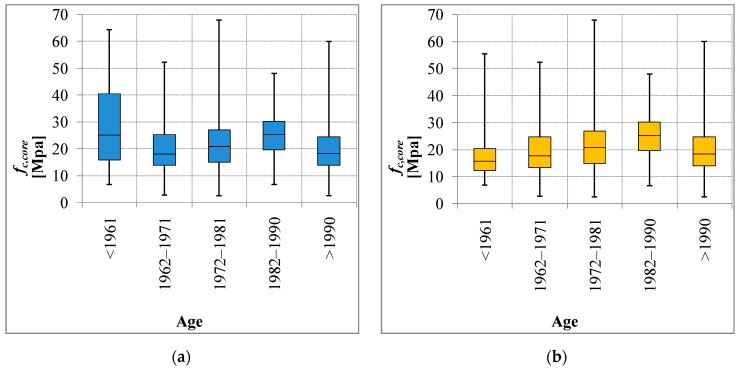
*f_c,core_*—Age comparison with (**a**) or without (**b**) civil engineering works. Each box encloses 50% of the data with the median value of the maximum *f_c,core_* displayed as a thin line, within the box; the top and the bottom of the box, respectively, mark the 25% and 75% limits of the population; outside the box, the whiskers represent the maximum and minimum values of the population, respectively.

Subset DB_1 considers all the results of the compression tests. It contains the data of the buildings and civil engineering works: 2010 samples in total. The results of the test show a significant variability of the compressive strength (*f_c,core_*). The first analysis shows the distribution in compressive strength classes of different design and construction periods. The analysis of the *f_c,core_* values indicates the variability given by the period under consideration. The mean values show an anomaly in the age group <1961 (Figure 5a) as a result of the presence of a significant group of data obtained from civil engineering works. For this type of construction, the design value of the concrete resistance was decidedly higher and there was also greater care in the construction phases.

Historical analyses of the code provision support the investigation. Based on the historical analysis of the original design approach, it is possible to avoid incorrect interpretations of the strength data and structural capacities. The RC construction works before 1972 were designed and built in accordance with the original and earliest Italian code in force at the time: Royal Decree no. 2229 of 1939. In this code, the design strength for concrete was the average compressive strength, ranging from 120 to 160 Kg/cm^2^. For structural elements subject only to axial load, the design strength was 180 Kg/cm^2^. These values were commonly considered for buildings. For civil engineering works, the typical design strength was up to 225 Kg/cm^2^. The above provision explained the high value of Figure 5a and then the value of the expected concrete compressive strength following the code in force at the time. In Figure 5b, the experimental data show a compressive strength consistent with the period. Since 1972, the design resistance was the characteristic strength of concrete R_ck_ which was not less than 150 Kg/cm^2^; in design practice, the most common values were not lower than 250 Kg/cm^2^. Thus the expected average values resulting from the tests should be higher than those of the previous period. Five periods were considered and summarized in Table 1, based on the design code and available data. 

### 3.2. Subset DB_2: Compression Test and Specific Weight

**Figure 6:**
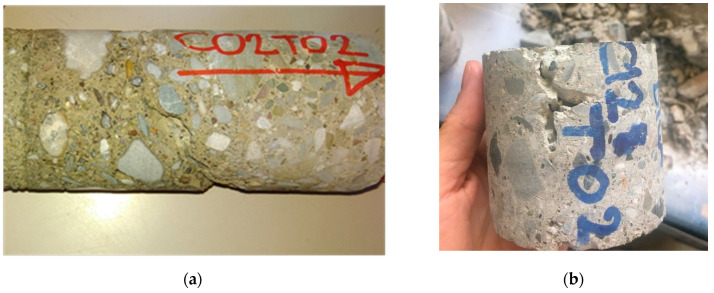
High porosity concrete in extracted samples: (**a**) Column, (**b**) Beam.

The analysis of the concrete specific weight (SW) is possible for a subset of 1039 samples.The specific weight depends on the water/cement ratio, aggregate/cement ratio, type of aggregate, original manufacturing procedures, and age.It can be considered to be representative of the global concrete quality: high porosity is generally associated with a lower strength of the structural elements. Therefore, SW could provide indications on performance and durability (which can affect the retrofitting strategies).In particular, the analyses look for the correlation between SW and compressive strength. Moreover, high porosity can affect the result of the compression test, as shown in Figure 6. Based on available data, four periods, summarized in Table 2, were considered, always based on the design code and available data.

**Figure 7:**
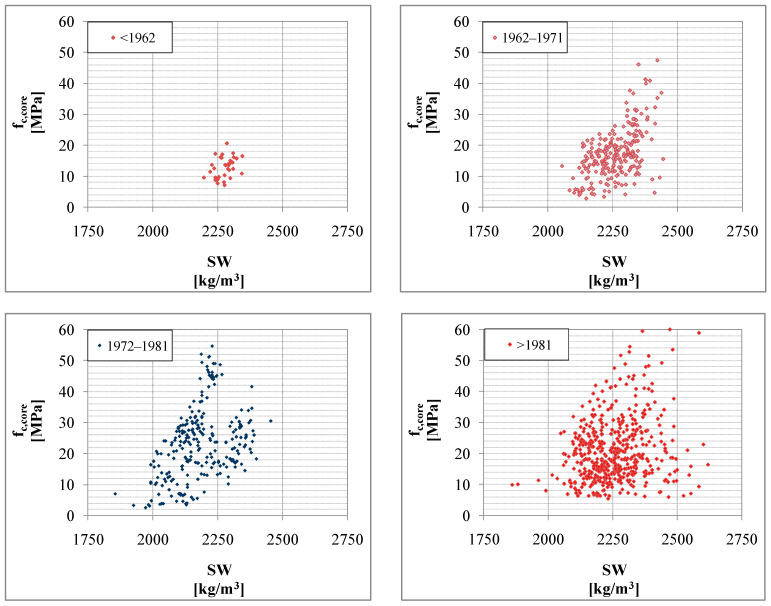
Correlation between Specific Weight (SW) and Compression Resistance of the samples, for different age of concrete based on the design code and available data.

Furthermore, the SW—*f_c,core_* relationship is analyzed. Some SW values seem to be of critical value for compressive strength and, in some cases (on single buildings), it is possible to identify a clear correlation between SW and compressive strength. However, contrary to expectations, there is no clear and generalizable relationship between SW and compressive strength, as is reported in Figure 7 for different age of concrete based on the design code and available data.

### 3.3. Subset DB_3: Compression Test, Rebound Hammer Test, Ultrasonic Velocity Test

**Figure 8:**
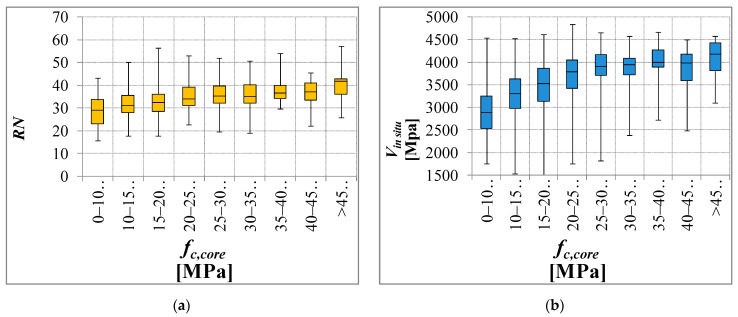
Box plot of NDT variability for several compressive strength classes: rebound number (**a**) and ultrasonic velocity tests (**b**).

The DB_3 subset considers all cases (1175 samples, 80 buildings) in which *f_c,core_*, *RN* (rebound index), *V* (direct ultrasonic velocity test) are simultaneously available. Cases where ultrasonic velocity was evaluated through surface measurements were excluded. To identify a potential relationship between DTs and NDTs, the analysis of the results of this subset plays a key role. However, the analyses generally show a poor or no correlation between the compressive strength and the considered NDTs. This result is particularly clear for the hammer tests (rebound number). The variability of the in situ compressive strength can be much greater than that shown by the NDTs (Figure 8).

**Figure 9:**
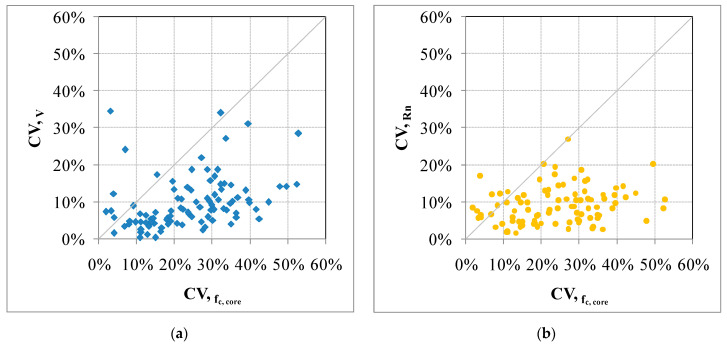
Relationship between DTs–NDTs within each building: rebound number (**a**) and ultrasonic velocity (**b**) tests.

Moreover, the relationship between NDT variability and DT variability for each construction was analyzed. A total of 80 buildings were analyzed and the results are shown in Figure 9. The variability of the DTs is not described by a similar variability of the NDTs, especially for the rebound number. In particular, the results of the rebound test show a poor correlation with concrete strength. Moreover, independently from the concrete strength, for the rebound test, the dispersion of the data is very high. The trend growth is significantly lower or negligible. NDTs show a limited variability which is intrinsic to the method and does not represent quality but an inability to represent the true characteristics of the concrete. In many cases, the coefficient of variation (CV) of the DTs is about 30% higher. However, this data is not indicative of problems within the data or that the investigation is not controlled but simply describes different concrete homogeneous areas. Therefore, in actual buildings, it seems unreliable to identify a relationship between NDTs and DTs. Looking at these results, identifying one or more concrete homogeneous areas based on the NDTs also appears to be difficult and unreliable.

For NDTs, considering the methods described in the previous paragraphs (in particular the combined SonReb method), it is believed that it is more reliable to use only the results of the ultrasonic tests to build a calibrated relationship between DTs and NDTs. This result is consistent with other recent studies (for example [51]) which, however, are based only on an experimental laboratory program (samples made in the laboratory). Derived from the exponential form SonReb, the calibrated expression could therefore be:

(2) $$f_{c}=a\cdot V_{}^{b}$$

where *a* and *b* are defined based on the DT and NDT results. In this way, it is also possible to consider a lower number of in situ tests, increasing the reliability of the tests and thereby reducing costs.The method can be effectively applied with the correction reported in the next paragraph.

### 3.4. Subset DB_4: Compression Test and Ultrasonic Velocity Test

**Figure 10:**
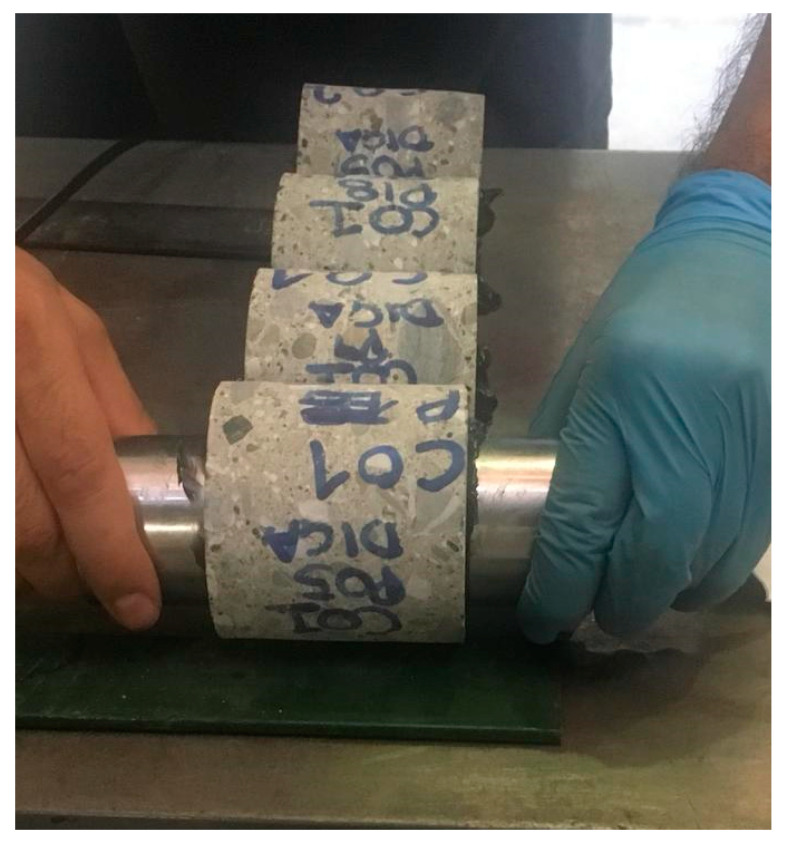
Direct measurement of ultrasonic velocity on single concrete samples.

**Figure 11:**
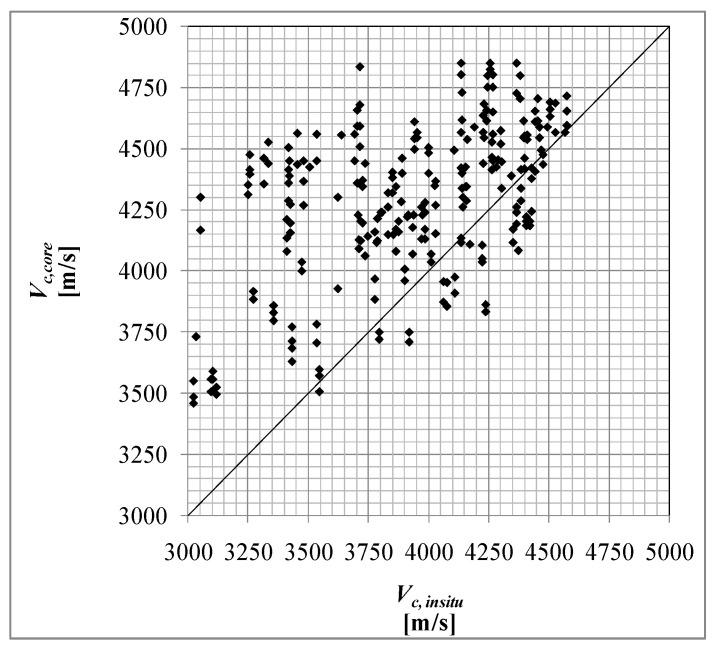
In situ direct ultrasonic velocity (*V_c,insitu_*) test vs. ultrasonic velocity test samples (*V_c,core_*).

Subset DB_4 reports the ultrasonic velocity as NDTs. DB_4 was defined considering only a subset of samples with a direct velocity measurement both on the structural elements (in situ) and on the single extracted samples (in the same points) before the compression test (Figure 10). In total, 45 data triples (*V_c,core_*, *V_c,insitu_*, *f_c,core_*) were obtained from an experiment conducted in the laboratory of the University of Basilicata [48] on fourbeams extracted from an existing and demolished RC school building. In addition, 202 homogeneous triples were also considered, divided into five homogeneous groups relating to 18 school buildings subjected to seismic assessment. The grouping considered in the following was defined by considering that the buildings belong to school complexes as well as their homogeneity (age and design code); the structural elements considered are mainly columns. Another 202 data triples (*V_c,core_*, *V_c,insitu_*, *f_c,core_*)were obtained. All these values are subsequently compared and Figure 11 reports the result of the first comparison.

As expected, in Figure 11, the in situ ultrasonic velocitiessignificantly underestimate the “true velocity” values obtained from the samples. This difference could be influenced by the surface degradation of the in situ concrete.

**Figure 12:**
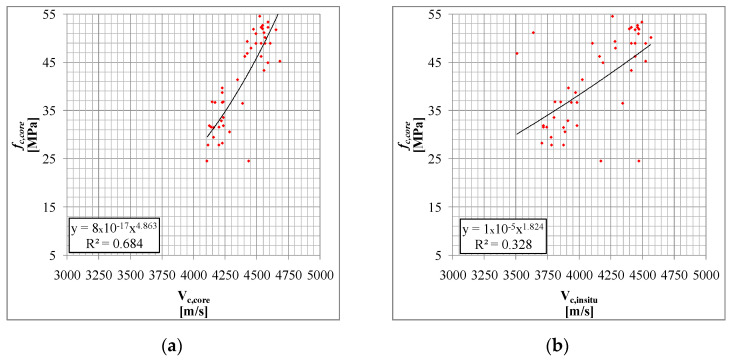
Comparisonof direct ultrasonic velocity samples vs. compressive concrete strength (**a**) and in situ ultrasonic velocity vs. compressive concrete strength (**b**).

In Figure 12, the results of the ultrasonic velocity NDTs are compared with the result of the compression tests. In Figure 12, 45 data triples of the University of Basilicata experiment [48] are reported, considering the relationships between *V_c,core_*-*f_c,core_* and *V_c,insitu_-f_c,core_* in Figure 12a whereas in Figure 12b; the best fit of the data is reported.

**Figure 13:**
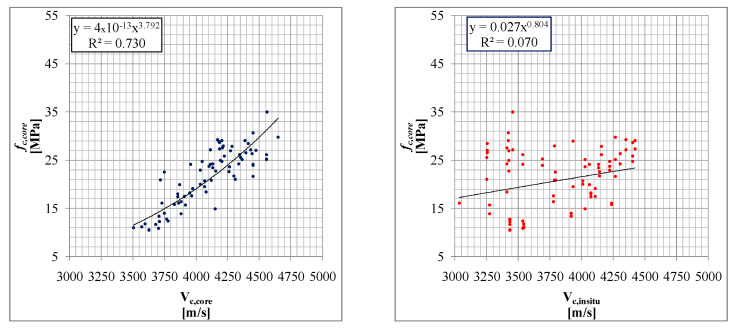

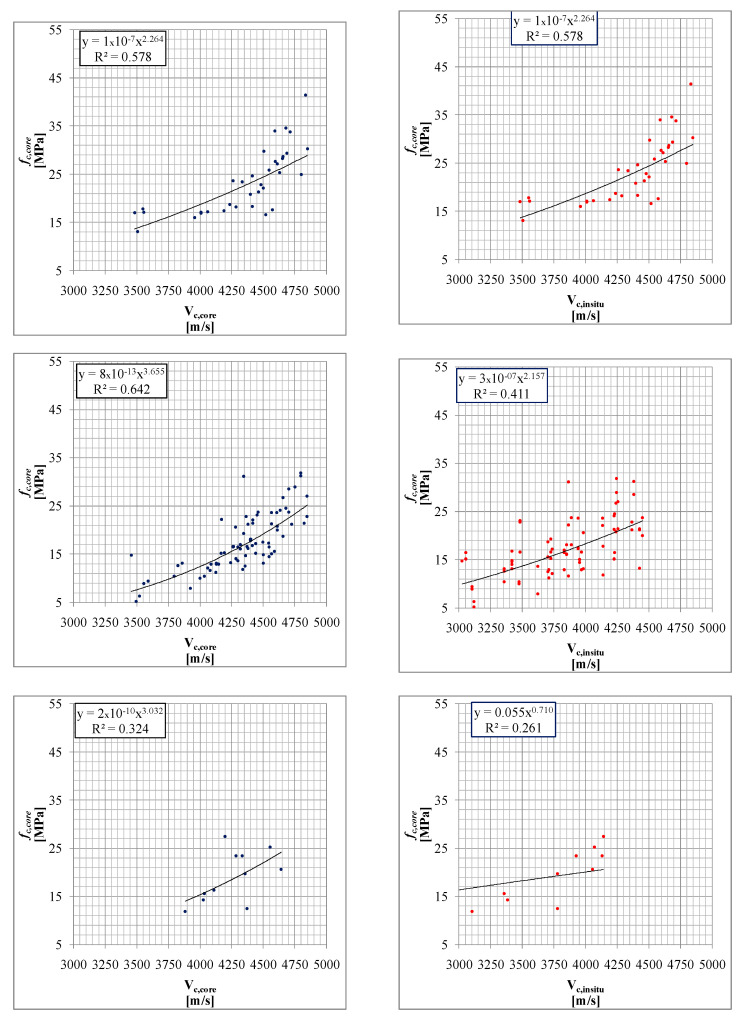

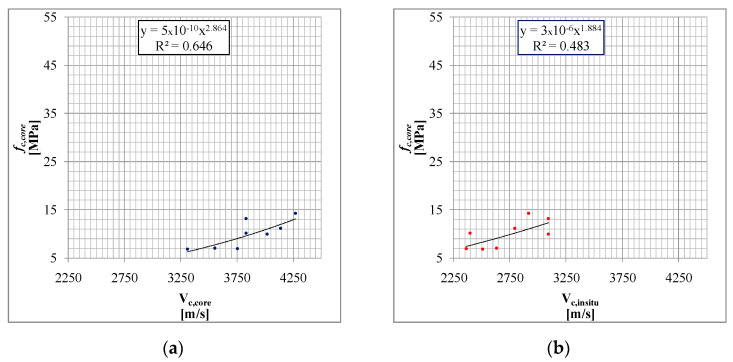
Ultrasonic velocity samples vs. compressive concrete strength (**a**) and in situ ultrasonic velocity vs. compressive concrete strength (**b**). The comparisons are related to the various samples taken in situ at different constructions and sites.

In Figure 13a, for those buildings under investigation, the relationships between *V_c,core_*-*f_c,core_* are reported; whereas in Figure 13b, for those buildings under investigation, the relationships *V_c,insitu_*-*f_c,core_* are reported; the best fit of the data is reported. In the first case, the internal variability of the ultrasonic method is very limited.

It is heavily influenced by the material and by the context [48] and it seems obvious that for in situ tests the measurement errors are systematic, and difficult to either estimate or eliminate. On the other hand, even in cases of high variability in *f_c,core_*,*V_c,core_*seems able to correctly estimate the *f_c,core_* values and follow its evolution. The effects of age and compressive stress, also referred to in several studies, seem less evident in the *V_c,core_*-*f_c,core_* relationship whereas for the *V_c,insitu_*-*f_c,core_* relationship, age also has a clear influence on the deterioration of the concrete and subsequent reduction of the reliability of the correlation.

The above-illustrated results show the ability of the ultrasonic tests to evaluate the concrete strength if the ultrasonic velocity measurement is carried out directly on the sample. Therefore, in situ ultrasonic tests could be used to estimate the compressive concrete strength by applying the expression reported in the previous paragraph, in which, however, the in situ ultrasonic velocity values are corrected with those measured on the extracted sample by adopting, for example, an average correction value given by the ratio between *V_c,core_* and *V_c,insitu_*. This proposal is also consistent with other studies (e.g., [12,15]).

### 3.5. Discussion

In the literature and previous studies, methods based on the NDT–DT relationship are considered reliable and are considered for applications also on existing RC constructions. As a matter of fact, as reported in the previous sections, in the last few years, for existing RC construction the investigation procedures based on NDTs and the commonly used NDT–DT correlations have shown significant critical issues, particularly in their ability to investigate and estimate the characteristics of the concrete. Such methods, which are often developed in laboratories and validated for new concrete, are often not suitable for investigating the existing RC constructions.

On the contrary, this study shows a significant degree of uncertainty, operational difficulties and potential errors in interpreting the results; they are based on experimental experiences on existing RC constructions. Based on the reported experimental campaigns, the critical issues in current NDT–DT relationships are clearly shown and some possible improvements are consequently defined from the obtained results (Section 3.3. and Section 3). For this reason, the data shown below can be considered an excellent reference to estimate the reliability of the strength values of concrete, based on past experience or the existence of similar structures. The availability of new additional data and the criticalities in practical professional applications allow for updating the procedures and evaluating their reliability.

## 4. Conclusions

In the last few years, the characteristics of concrete have been investigated using empirical expressions in order to evaluate the in situ compressive strength using NDTs, namely the rebound hammer test and ultrasonic pulse velocity test. In other studies, the variability of the most common NDT (rebound hammer test and ultrasonic pulse velocity test) was analyzed [12,13,52,53]. Generally, the variability within these methods is very small.

The present study reported data and procedures, based on a database of real data. A new contribution from the study is really the database. The database was obtained from laboratory investigations and professional practice activities. On the contrary, several interesting and recent studies are developed in laboratories and validated only for new concrete where the results do not take into account the quality of the concrete in existing reinforced concrete constructions and its low strength and degradation problems.

However, based on the results of this study, it can be stated that many of the existing empirical methods (based on pre-established correlations) are unable to provide a reliable evaluation of the compressive concrete strength and its variability.

In particular, the results obtained show variability in compressive concrete strength but highlighted the non-variability of the Rebound Number (RN) and its non-correlation with concrete compressive strength. Ultrasonic velocity shows an extremely limited variability although it can be increased by relating the in situ ultrasonic velocity and the ultrasonic velocity samples.

In conclusion, based on this work, it can be highlighted that:

1. The rebound hammer test is not representative of the compressive concrete strength and is also misleading. It must be excluded. The classic SonReb method should not be used;
2. Ultrasonic velocity tests can be used only if suitably calibrated with Ultrasonic velocity tests on the extracted concrete before the compression tests;
3. Using current methods and procedures, NDTs cannot be used a priori to identify homogeneous areas.

Lastly, this study contains the experimental data useful as a reference both for practical professional activities and for the comparison and validation results used in research activities [54].

This study allows for further developments due to:

– An increase in the amount of experimental data available;
– The improvement of data collection procedures and standardization of analysis procedures;
– The updated indication in the codes and guidelines.

In the next step of the study, new procedures and methods will be developed based on the reported results, in particular, following the results of Section 3.4; the new research trend based on ANNs can also be applied using the real database, suitably treated and analyzed. The strengths and mechanical characteristics of concrete play a key role in the safety levels of construction. These issues were often underestimated but represent one of the most important issues to define optimal retrofitting strategies [55].

## Figures and Tables

**Table 1:** DB_1: composition and main statistical values.

		*f_c,core_*			
Construction Age	n° Samples	Mean Value [MPa]	Deviation Standard [MPa]	Min [MPa]	Max [MPa]
<1961	256	28.24	14.73	6.77	64.27
1962–1971	416	19.77	9.00	2.76	52.21
1972–1981	507	21.94	11.01	2.55	67.87
1982–1990	64	25.97	9.59	6.72	47.96
>1990	767	20.27	9.12	2.48	59.90
Total	2010				

**Table 2:** DB_2: composition and main statistical values.

Construction Age	n° Samples	Percentage of Samples	Specific Weight Mean Value [kg/m^3^]	Deviation Standard [kg/m^3^]	Min [kg/m^3^]	Max [kg/m^3^]
<1962	35	3.37%	2277	34	2197	2345
1962–1971	248	23.87%	2260	79	2056	2444
1972–1981	265	25.51%	2194	110	1854	2455
>1981	491	47.26%	2257	112	1860	2619
	1039					

## Data Availability

Not applicable.
